# Severe bleeding in patients following “tubeless” percutaneous nephrolithotomy: Predictors of angioembolization

**DOI:** 10.1177/03915603241282409

**Published:** 2024-09-30

**Authors:** K. R. Surag, Abhijit Shah, Kasi Vishwanath Gali, A. V. B. Krishnakanth, Arun Chawla, Padmaraj Hegde, Anupam Choudhary, Mithun Rao

**Affiliations:** 1 Kasturba Medical College, Manipal, Manipal Academy of Higher Education, Manipal, Karnataka, India; 2 Kasturba Medical College, Mangalore, Manipal Academy of Higher Education, Manipal, Karnataka, India

**Keywords:** Bleeding, hemorrhage, angioembolization, pseudoaneurysm, arteriovenous fistula, coils, creatinine, PCNL, tubeless

## Abstract

**Introduction:**

Percutaneous nephrolithotomy (PCNL) is a widely used procedure for treating renal calculi. Advanced techniques have improved outcomes, but hemorrhage remains a significant complication. While most cases of hemorrhagic complications are typically managed conservatively, few cases necessitate interventions like angioembolization (AE). The purpose of this study is to identify risk factors closely associated with severe bleeding post-PCNL requiring AE and to assess if these factors can independently predict the type of lesion [arteriovenous fistula (AVF) vs pseudoaneurysm (PA)].

**Materials and method:**

A retrospective analysis was conducted on 119 patients who underwent “tubeless” PCNL and experienced severe bleeding between January 2018 and December 2023. The study reviewed demographic characteristics, stone characteristics, perioperative factors, and adverse events. The chi-square test and Fisher’s exact test were used for univariate analysis. Logistic regression analysis was used in binomial analysis with a value of *p* < 0.05 considered statistically significant.

**Results:**

Out of 119 patients, 51 required AE. Elevated preoperative serum creatinine levels (>1.5 mg/dl) [*p* = 0.01], upper pole access [*p* = 0.008], and a larger access sheath size (standard PCNL vs mini-PCNL) [*p* ⩽ 0.001] were found to be significantly associated with AE. Logistic regression analysis revealed standard PCNL was significantly associated with post-PCNL bleeding requiring AE (odds ratio [OR]: 50, 95% confidence interval [CI]: 6.529–382.90, *p* ⩽ 0.001). Stone size and co-morbidities showed no significant association with AE. The average duration of presentation of symptoms post PCNL was 13.6 days. Most patients underwent coiling for AE, with a clinical success rate of 94%.

**Conclusion:**

Elevated serum creatinine levels, upper pole access, and tract size >24 Fr are more prone to post-tubeless PCNL severe bleeding, which requires renal AE. The findings suggest that early angiography and possible AE should be considered for at-risk patients. In the future, these predictors may be integrated into predictive models to improve patient risk stratification.

## Introduction

PCNL (percutaneous nephrolithotomy) has withstood the test of time as the go-to modality for renal stones, especially those more than 2 cm.^1^ The miniaturization of instruments with a superior understanding of the role of patient, stone, and operative factors of PCNL has led to better outcomes in terms of stone clearance rates (>90%) and reduced complication rates (~7% major complications).^2,3^ Hemorrhage is a significant cause of concern in patients of PCNL, with the risk of arteriovenous fistula (AVF) or pseudoaneurysm (PA) formation requiring angioembolization (AE) being 1%.^4^

Hemorrhage can be intraoperative or postoperative. The use of large tract size, multiple punctures, and excessive torque during the procedure can lead to intraoperative hemorrhage. Postoperative hemorrhage can be early or delayed. Bleeding from the nephrostomy tube, hematuria, and perinephric hemorrhage constitute early postoperative hemorrhage, which is usually managed conservatively. In contrast, delayed postoperative hemorrhage is most frequently due to AVF and PA, which require immediate attention. Although rare, its occurrence can be fatal if not identified and treated early. Most of the time, conservative treatment is sufficient to control the bleeding. However, in cases of failure of conservative management, angiography followed by AE is the procedure of choice.^5^

This retrospective study follows the data of those patients with severe bleeding post-“tubeless” PCNL and aims to understand better the predictive factors of post-PCNL severe bleeding eventually requiring AE. We also compared these predictive factors with AVF and PA separately.

## Materials and methods

### Study design, settings, and participants

This retrospective single-center study performed at the Department of Urology and Renal Transplant at Kasturba Medical College and Hospital reviews the data of 119 patients, of the 1012 total patients who underwent tubeless PCNL at our institute, over the past 5 years from January 2018 to December 2023 and who had prolonged hospital stays or were re-admitted because of severe bleeding. “Severe bleed” was defined as a fall in hemoglobin of 2 g % or recurrent hematuria not controlled with conservative methods for 10 days or hemodynamic instability with an increasing perinephric hematoma requiring urgent stabilization with fluids, blood products, and antibiotic coverage or need of blood products at any time in the postoperative period within the first month. If the serum creatinine was normal, the patient underwent contrast-enhanced computed tomography (CECT), whereas patients with elevated creatinine underwent ultrasound with Doppler imaging and non-contrast computed tomography (NCCT).

After reviewing the case records, those cases requiring AE were studied along with the type of vascular injury (PA, AVF, or both). The clinical success of AE is defined as the complete resolution of hematuria with stable laboratory parameter trends and no unplanned hospital visits within the first 30 days.

Cases of failed AE were also reviewed. Clinical failure was defined when there was a need for further endovascular and surgical intervention due to the recurrence of bleeding within a 30-day follow-up period.

All patients with intra-operatively recognized troublesome bleeding requiring nephrostomy tube placement were excluded from this study. All incomplete data records were also excluded from the present study.

### Percutaneous nephrolithotomy

All patients underwent percutaneous puncture in a prone position under fluoroscopic guidance with either standard (24–34 Fr) or mini-PCNL (12–18 Fr) as per surgeons’ preference. Wolf nephroscope (12 Fr/Dresden or 20 Fr) was used with a pneumatic lithotripter (Swiss Lithoclast) or Ho:YAG/Thulium fiber Laser. On completion of the procedure, all patients had double-J stent placement. We do not routinely place nephrostomy tubes. Nephrostomy tubes are placed in cases of intra-operative bleeding or infected systems. A urethral catheter is removed on postoperative day (POD) 1, and discharge is planned for POD 2.

### Angioembolization

In cases of severe bleeding, patients underwent AE after angiography. All procedures were performed by the interventional radiology department with the right common femoral artery as vascular access. After selective renal catheterization, 5–10 cc of contrast is injected to locate the site of active extravasation. Other findings include the presence of PA and AVF. AE was performed with either coiling (Nestar) or glue (NBCA/Histocryl). Technically, it was declared successful when the complete exclusion of the treated vessel was noted on repeat angiography.

### Data collection, variables, and statistical analysis

All data was collected after searching medical records, including patient, stone, and operative factors. Data on AE methods and vascular injury were also noted. Preoperative serum creatinine was categorized into less than 1.5 mg/dl and more than 1.5 mg/dl. The stone sizes were taken up to the longest diameter in millimeters. PCNL was divided into standard PCNL (24 Fr and above) and mini PCNL (< 20 Fr). Jamovi application is used as the graphical user interface of R language programing for statistical analysis.^6^ The chi-square test and Fisher’s exact test were used for univariate analysis. Multivariate analysis was performed using logistic regression with a significance level of *p* < 0.05.

## Results

During the duration of our study, a total of 1012 patients underwent tubeless PCNL at our institute, of which 119 patients presented with severe bleeding post-PCNL. 51 patients out of 119 required AE. Mean (range) age of the study group was 48 years (23–79). Of the total patients recruited in the study with severe bleeding, males represent approximately 3.5 times more (*n* = 93) than females (*n* = 26). A comparative analysis of the angioembolized group and the group managed conservatively is described in Table 1. The angioembolization population is almost 6.5 times more males (*n* = 44) as compared to females (*n* = 7) with an odds ratio of 2.44 (95% CI: 0.936–6.35; *p* = 0.063). Those patients who underwent angioembolization were compared to those managed conservatively. The mean age was comparable in both groups with severe bleeding (*p* = 0.437). The demographic and PCNL-related factors are mentioned. Significant factors between the two groups were pre-operative serum creatinine levels (>1.5 mg/dl) (*p* = 0.01), upper pole access (*p* = 0.08), and larger access sheath size (standard PCNL vs mini-PCNL) (*p* ⩽ 0.001). On binomial regression analysis, standard PCNL was significantly associated with requiring AE post-PCNL bleeding. (odds ratio [OR]: 50, 95% confidence interval [CI]: 6.529–382.90, *p* ⩽ 0.001). Based on the characterization of complications, 52 patients were managed with IV fluids alone (Clavien-Dindo classification - Grade I), 16 warranted blood transfusion (Grade II) and 51 required angioembolization (Grade IIIb)^7,8^

**Table 1.** Demographic and PCNL-related factors.

Factors	Patients (*n* = 119)		Odds ratio (95% confidence interval)	*p*-Value
	Requiring angioembolization (51)	Managed conservatively (68)		
Age (Mean ± SD)^a^	51 ± 13.6	68 ± 14.4		0.43
Diabetes^a^	15	20	1.00(0.45–2.22)	1
Hypertension^a^	22	20	1.82 (0.85–3.90)	0.12
Ischemic heart disease^a^	3	2	2.06 (0.332–12.8)	0.42
Antiplatelets/Anticoagulants	9	8	1.61 (0.573–4.51)	0.36
**Pre-op serum Creatinine** **(>1.5 mg/dl)^a^**	**15**	**8**	**3.13 (1.21–8.10)**	**0.01**
Previous surgery on ipsilateral renal unit^a^	2	8	0.306( 0.062–1.51)	0.12
Stone volume (mm^3^)^b^	4063	2623	–	0.21
Staghorn stone	9	22	2.23 (0.93–5.39)	0.07
Density of stone (HU value)^b^	1004	934	–	0.30
Side	Left—23	Left—40	1.74 (0.836–3.62)	0.13
	Right—28	Right—28		
Multiple access tracts	6	8	1.00 (0.324–3.09)	1
**Standard PCNL (24** **Fr and above)**^a^	**50**	**34**	**50 (6.53**–**383)**	<**< 0.001**
**Mini-PCNL (20** **Fr and below)**^a^	**1**	**34**		
Mean operative time^b^	46 min	47 min		0.8
**PCNL access (pole)**				
(Upper)	5	0	16.2 (0.87–300)	0.008
(Middle)	1	50	1.52 (0.134–17.2)	0.73
(Lower)	13	38	0.564 (0.229–1.39)	0.21
Nephrostomy tube placement^a^	7	7	1.39 (0.454–4.24)	0.56

a Chi-square test of independence.

b Mann-Whitney *U* test.

c Fischer exact *t*-test.

Bold value represents less than 0.05 considered significant.

Of the 51 patients requiring AE (Table 2), the mean number of days for presentation post-PCNL with severe bleeding was 13.6 days (ranging from day 4 to day 38 post-procedure). They had varied presentations: patients presenting early either had un-resolving hematuria or hemodynamic instability, while those presenting later had clot retention, flank pain, and persistent hematuria. Each patient was admitted and had a prolonged hospital stay (~5.8 ± 5 days).

**Table 2.** Characteristics of angioembolization.

Factors	*n* (%)
Characteristics of lesions	
AVF	10 (20)
PA	39 (76)
Both	2 (4)
Clinical success	48 (94)
Failure	3 (5.9)
Death	2* (3.9)

Of the three cases that failed, one patient died due to overwhelming blood loss, one patient underwent simple nephrectomy, and one required partial nephrectomy.

* One patient successfully embolized but died due to COVID-19.

In angiographic studies, 39 patients had developed PA, whereas ten patients had an isolated AVF (Table 3). Only two patients had both or multiple lesions. Of the 51 patients, 48 were declared a clinical success and discharged after the same. Our analysis demonstrates AVF and PA groups have mean ages of 47.9 ± 14.6 and 50.4 ± 13.6 years, respectively. The difference in age between the two groups was not statistically significant (*p* = 0.63). The gender distribution in the two groups was also not significantly different (male/female: 6:3 in AVF and 36:4 in PA; *p* = 0.07). Additionally, none of the selected factors showed a significant difference between the two groups of AVF and PA.

**Table 3.** Characteristics of the study population based on either complication.

Factors	Patients (*n* = 49)		Odds ratio (95% confidence interval)	*p*-Value*
	AVF^9^	PA (40)		
Age (Mean ± SD)	47.9 (±14.6)	50.4 (±13.6)		0.63
Sex (Male)	6	36	4.50 (0.799–25.3)	0.07
(Female)	3	4		
History of diabetes	2	11	1.33 (0.238–7.40)	0.74
History of hypertension	3	17	1.48 (0.323–6.77)	0.61
History of ischemic heart disease	0	1	0.722 (0.0272–19.1)	0.63
Pre-op Sr Creatinine (>1.5 mg/dl)	1	12	3.43 (0.385–30.5)	0.24
Staghorn stone	1	8	0.500 (0.054–4.60)	0.53
Side (right)	3	23	2.71 (0.591–12.4)	0.18
Multiple access tracts	5	1	0.875 (0.089–8.56)	0.9
Standard PCNL (24 Fr and above)	38	9	1.42 (0.05–37.7)	0.63
Mini-PCNL (20 Fr and below)	1	0		
Mean operative time (minutes)	42.6	47.3		0.27
Previous surgical history on the same side	0	2	1.23 (0.054–27.9)	0.49
PCNL access (pole)				
(Upper)	0	5	2.94 (0.149–58)	0.26
(Middle)	None	None	–	–
(Lower)	4	8	3.20 (0.696–14.7)	0.12
Nephrostomy tube placement	2	3	0.284 (0.039–2.02)	0.18

* Pearson Chi-square/Fischer’s exact *t*-test or independent sample *t*-test.

Three cases were declared clinical failures. One patient died due to overwhelming blood loss. One patient, after the failed procedure, underwent a partial nephrectomy, whereas one patient underwent a simple nephrectomy. One patient declared a clinical success, died on post-procedure day 6 in the ward from complications of COVID-19. None of the patients showed signs of post-embolization syndrome. None of the patients required additional blood products post-embolization procedure.

All 119 patients’ follow-up data was recorded at third month, as is our institutional protocol in complicated PCNL outcomes. Only patients who underwent surgical exploration had significantly improved Serum creatinine levels as compared to their discharge values. However, only those patients who underwent AE were directed to follow up at the end of sixth month for imaging and serum creatinine levels. Of the 51 patients, 42 patients followed up, and 9 were lost to follow-up. In these 42 patients, there were no significant differences in their serum creatinine levels as seen during their discharge.

## Discussion

PCNL is a grade IV controlled renal trauma and, as such, is associated with varying amounts of blood loss during puncture, tract dilatation, and stone disintegration.^9^ The overall incidence of blood transfusion rates post-PCNL has been quoted as 0.8%–17.5% in literature.^2,10^ The arterial damage leads to the subsequent development of AVF or PA. Injury to the high-pressure system (segmental arteries) causes a leak into either the low-pressure venous system, leading to the formation of AVF, or the parenchyma and hilar tissue, leading to the formation of PA.^11^

Three significant factors that were strongly associated with patients requiring AE post-PCNL bleeding were raised serum creatinine levels (>1.5 mg/dl) preoperatively, upper pole access, and increased tract size for patients undergoing PCNL (24 Fr and above). There are not many studies that associate pre-operative serum creatinine levels with troublesome bleeding. Zehri et al.^12^ demonstrated in their retrospective study the female gender, along with the history of chronic kidney disease, is a strong trigger factor for the need for blood transfusion post-PCNL. Elevated serum creatinine levels hamper efficient platelet functioning and altered interaction between endothelium and platelets. However, many studies have found contrasting evidence where these findings were not supported.^13–15^ A study by Arora et al.^15^ demonstrates the role of stone complexity, number of access tracts, pelvicalyceal injury, and history of prior surgery to be significant factors, which contrasts with the findings in the current study.

El-Nahas et al.^16^ reported factors strongly associated with significant bleeding post-PCNL, that is, upper calyceal entry, solitary kidney, staghorn calculi, multiple punctures, and the surgeon’s experience level. In our study, upper-pole access was significantly associated with the need for AE. Upper pole access may lead to a higher chance of posterior segmental arterial injury, unlike a lower pole approach, which leads to our finding. Jinga et al.^17^ conducted a similar study to predict factors for embolization post-PCNL, where upper calyceal access was also a predictor for the same. However, it is well known that not only does the calyx of entry hold importance, but so does the tract trajectory. The more oblique the tract, the more it traverses the parenchyma and blood vessels, causing excess bleeding.^18^ A strong emphasis is noted on the correctness of puncture in the study by Kim et al.^19^

The advent of miniaturization of PCNL instruments has led to a significant drop in blood-loss incidence post-PCNL.^20–22^ Our study also demonstrates the impact of miniaturization on the need for AE—a smaller access tract causes lesser parenchyma trauma and a better chance for spontaneous resolution through internal tamponade.

Unlike many other studies, stone size, operative time, laterality, diabetes, hypertension, staghorn calculi, and the number of access tracts did not show a significant difference in the number of patients eventually requiring AE. Various studies have been summarized in Table 4, which contrast our current study findings like the results of Irani et al.^17^ and Un et al.^23^ where renal anomalies, diabetes, and size of stones are significant parameters for predicting angioembolization in these patients and Shin et al.^24^ which discovered significantly more bleeding in patients enduring longer operative times.

**Table 4.** Summarized results of the present study and other studies in the literature on angioembolization post-PCNL.

S. No	Study	Type	No. of pt require AE	Salient factors
1	Arora et al.^15^	Retrospective single center	9	H/o previous renal surgery, Stone complexity, Number of access tracts, Pelvicalyceal injury
2	Irani et al.^5^	Retrospective single center	72	Diabetes, Renal Anomaly
3	Kim et al.^19^	Retrospective single center	21	Correctness of puncture
4	Un et al.^23^	Retrospective single center	14	Renal anomalies, mean size of stones
5	El-Nahas et al.^16^	Retrospective single center	39	Upper calyceal access, solitary kidney, staghorn calculi, multiple punctures, Inexperience surgeon
6	Shin et al.^24^	Retrospective single center	10	Mean Operating time was significant for bleeding (could not calculate for need of AE)
7	Present study	Retrospective single center	51	Pre-operative serum Creatinine, Upper pole access, Increased access tract size

All 51 patients underwent super-selective AE, as this causes the least amount of parenchymal loss. Of the 51 patients, 47 (92%) underwent coiling, and the rest underwent glue instillation (8%) in view of certain resource or financial constraints. Pushable or detachable coils are universally used, as seen in our institute, with occasional glue usage.^25–27^ The clinical success rate of AE in our study (94%) was significantly higher than those seen in some of the other studies^25,27^

In our study, the mean number of days for presentation post-PCNL with severe bleeding was 13.6 days, ranging from day 4 to day 38 post-procedure. Arora et al.,^11^ Nouralizadeh et al.,^15^ and Srivastava et al.^28^ had earlier mean presentation days (fifth–eighth day).

The limitations of our research include possible selection bias due to its retrospective nature and the fact that it was conducted at a single center, which may limit the generalizability of our findings. The nature of this complication is not common, hence the retrospective nature of these studies. Our sample size is small, which may obscure an accurate representation of the data analysis. In the future, combining the data at a multi-institutional level may help bridge this limitation. The usage of mini-PCNL versus standard PCNL varied from surgeon to surgeon, which may also alter our findings and analysis. There are gaps in data that present some variables like stone nephrolithometry scoring and more technical details on the method of AE.

## Conclusion

In conclusion, our study underscores the critical importance of high-risk factors for severe bleeding in post-PCNL. Elevated serum creatinine levels, upper pole access, and larger access sheath size emerged as significant predictors of this complication, necessitating early angiography and potential AE for optimal patient management. Future prospective studies can be done to incorporate these risk factors as part of the nomogram to predict bleeding risk leading to AE. By doing so, we can effectively reduce morbidity associated with severe bleeding post-PCNL and pave the way for better outcomes in this patient population.
